# Elevator Forceps

**Published:** 1858-07

**Authors:** James Bate

**Affiliations:** 47, Old Steine, Brighton


### ARTICLE XVIII.

### *Elevator Forceps.*

We all know, from the formation of the fangs of the wisdom teeth, in the under jaw, the difficulty that exists in their extraction; I therefore, without any apology, forward you some account of a pair of favorite forceps I use for that purpose. I term them Double Elevators, and the manner in which they are used is as follows: the blades are opened, and the instrument passed into the mouth, with one blade on either side of the tooth, and immediately on reaching the posterior side of the second molar, the hand is closed tightly and firmly, which of course closes the beak, and consequently forces the instrument between the second and third molars, at the same time starting the tooth, and then, by simply depressing the handles, and making a fulcrum of the second molar, the tooth is raised out of its socket, in the direction of the fangs, and with considerably less pain than with the ordinary elevator. The blades of the instrument are difficult to describe; they are knife-edged, bent at a slight angle, and made thin as possible in compatibility with strength, and the handles slightly curved to admit of the instrument's clearing the under teeth. I have never seen a pair like them, save in my father's surgery, and they are new to all to whom I have shown them.

James Bate.

47, *Old Steine, Brighton, January,* 1858.

[*Lon. Quar. Jour. Den. Sci.*

---

[These forceps were invented and used by Dr. L. S. Parmly, of New Orleans, more than thirty-five years ago. They have also been used by many other practitioners in the United States, and are regarded by some as very valuable. The use of them, however, has never become very general.—Eds.]

